# Neural Substrates of Sexual Desire in Individuals with Problematic Hypersexual Behavior

**DOI:** 10.3389/fnbeh.2015.00321

**Published:** 2015-11-30

**Authors:** Ji-Woo Seok, Jin-Hun Sohn

**Affiliations:** Department of Psychology, Brain Research Institute, Chungnam National UniversityDaejeon, South Korea

**Keywords:** problematic hypersexual behavior, sexual desire, functional magnetic resonance imaging, dorsolateral prefrontal cortex, hemodynamic response

## Abstract

Studies on the characteristics of individuals with hypersexual disorder have been accumulating due to increasing concerns about problematic hypersexual behavior (PHB). Currently, relatively little is known about the underlying behavioral and neural mechanisms of sexual desire. Our study aimed to investigate the neural correlates of sexual desire with event-related functional magnetic resonance imaging (fMRI). Twenty-three individuals with PHB and 22 age-matched healthy controls were scanned while they passively viewed sexual and nonsexual stimuli. The subjects' levels of sexual desire were assessed in response to each sexual stimulus. Relative to controls, individuals with PHB experienced more frequent and enhanced sexual desire during exposure to sexual stimuli. Greater activation was observed in the caudate nucleus, inferior parietal lobe, dorsal anterior cingulate gyrus, thalamus, and dorsolateral prefrontal cortex in the PHB group than in the control group. In addition, the hemodynamic patterns in the activated areas differed between the groups. Consistent with the findings of brain imaging studies of substance and behavior addiction, individuals with the behavioral characteristics of PHB and enhanced desire exhibited altered activation in the prefrontal cortex and subcortical regions. In conclusion, our results will help to characterize the behaviors and associated neural mechanisms of individuals with PHB.

## Introduction

Problematic hypersexual behavior (PHB) is defined as the continuous participation in repeated sex acts with no control over excessive sexual compulsivity and behavior despite the awareness of the associated negative outcomes (Goodman, 1993; Carnes, 2001, 2013). Those who suffer from PHB can experience extreme difficulties in their family relationships and job performance. In addition, they are at greater risk for contracting sexually transmitted diseases or experiencing unwanted pregnancies from promiscuous sexual relations (Schneider and Schneider, 1991; Kuzma and Black, 2008). In the US, 3–6% of the community and college students have PHB (Coleman, 1992; Black, 2000; Seegers, 2003). In Korea, approximately 2% of all college students have PHB (Kim and Kwak, 2011). Due to its high prevalence and related problems, the associated risks are increasingly recognized in society as the incidence of PHB appears to be growing.

Although the seriousness of PHB is now recognized, it was not included in the DSM-5 (American Psychiatric Association, 2013) Debates are ongoing as to whether hypersexual disorder should be classified as a disease; therefore, there is no consensus on its definition, classification, or diagnostic criteria. This reflects the difficulties in establishing a clear classification standard due to the lack of objective and empirical studies on the factors related to hypersexuality disorder.

Although, the classification of PHB as a disease is still controversial, it has been proposed that excessive sexual activity should be classified as a category of addictive disorders because PHB includes symptoms that are similar to other forms of addiction (Goodman, 2001; Kor et al., 2013). Enhanced desire is strongly related to the clinically relevant aspects of addictive disorders. Imaging studies have shown that the function of brain regions that are involved in desire is altered in those with substance addiction (Garavan et al., 2000; Tapert et al., 2003; Franklin et al., 2007; McClernon et al., 2009). Behavioral addictions, such as gambling, internet gaming, and sexual behavior, that do not involve the direct intake of drugs also involve a heightened desire that seems to be related to altered functions in relevant brain regions (Crockford et al., 2005; Ko et al., 2009; Kühn and Gallinat, 2014; Voon et al., 2014).

Brain imaging studies of desire in substance addiction and behavioral addiction have shown functional changes in the prefrontal cortex (PFC) and subcortical reward circuits in subjects with these disorders (Goldstein and Volkow, 2011). In particular, these studies have identified the key involvement of the PFC in addiction, both through its regulation of limbic reward regions and its involvement in the motivational aspects of repetitive substance use and compulsive behavior. The disrupted functioning of the PFC leads to impairments in response inhibition and salience attribution, such as the attribution of inappropriately excessive salience to an addictive cue, as in substance and addicted behaviors, and a decreased desire for normal rewarding stimuli (Goldman-Rakic and Leung, 2002; Goldstein and Volkow, 2011).

Consistent with these results, the results of a neuroimaging study on PHBs suggested that individuals with PHBs have greater subjective sexual desire compared to healthy controls and that the enhanced desire is associated with different patterns of neural responses in the dorsal anterior cingulate-ventral striatal-amygdala functional network (Voon et al., 2014). In a brain structure and functional connectivity study, Kühn and Gallinat (2014) demonstrated that frequent pornography exposure is associated with altered brain structure and functioning in PFC areas and might lead to a tendency to search for novel and more extreme sexual material.

These studies provide evidence that heightened desire and the functional abnormalities implicated in desire are also involved in PHB, even though the behavior itself does not induce neurotoxic effects.

Unfortunately, the empirical data on sexual desire-associated neural responses in individuals with PHB are insufficient. Previous studies on the brain mechanisms underlying the processing of sexual desire in individuals with PHB have used conventional block paradigms during functional magnetic resonance imaging (fMRI) and a relatively prolonged exposure to erotic stimuli. In studies of sexual desire, the presentation duration appears to be important from a methodological point of view and because of differences in information processing (Bühler et al., 2008). In block designs, the duration of stimulus presentation is prolonged, and the occurrence of continuous stimuli in a block is completely predictable (Zarahn et al., 1997). Therefore, block designs likely activate areas that are associated with cognitive processes, such as sustained attention, top-down control, and the inhibition of sexual arousal. This could lead to reduced emotional involvement and therefore change the underlying neural activity (Schafer et al., 2005). Methodologically, event-related designs are inferior to conventional block designs for detecting activated brain areas, while they are superior for estimating hemodynamic response function (Birn et al., 2002).

Therefore, the objectives of this study were to (1) replicate previous behavioral findings of heightened sexual desire in individuals with PHBs, (2) identify the changes in brain function in regions known to be associated with enhanced desire, and (3) understand the differences in the hemodynamic responses of those brain areas over time in individuals with PHBs by using event-related fMRI. We hypothesized that individuals with PHBs are more likely to show greater sexual desire compared to healthy controls and that brain regions, such as the PFC and subcortical reward circuits, show altered activity and hemodynamic responses compared to healthy controls.

## Methods

### Participants

The present study included 23 heterosexual male participants in the PHB group [mean age = 26.12, standard deviation (SD) = 4.11 years] and 22 heterosexual male participants in the control group (mean age = 26.27, *SD* = 3.39 years). Approximately 70 potential participants were recruited from treatment facilities for problematic sexual behavior and Sex Addiction Anonymous meetings. The inclusion criteria were based on the PHB diagnostic criteria of previous studies (Table S1; Carnes et al., 2010; Kafka, 2010). The exclusion criteria were the following: age over 45 or under 18; a serious psychiatric disorder, such as alcohol use disorder, gambling disorder, major depressive disorder, bipolar disorder, or obsessive-compulsive disorder; currently taking medication; a history of serious head injury; homosexuality; a criminal record; or ineligibility for imaging (i.e., having a metal in his body, severe astigmatism, or claustrophobia). The clinicians conducted clinical interviews of all of the potential subjects, and a final group of 23 males who met the inclusion criteria and not the exclusion criteria were selected for the PHB group. For the control group, 22 participants with demographic characteristics (age, gender, education level, and income level) that matched the PHB group were selected. All of the participants provided written informed consents after the contents of the present study were explained to them. The Chungnam National University Institutional Review Board approved the experimental and consent procedures (approval number: 201309-SB-003-01). All of the participants received financial compensation (150 dollars) for their participation.

### Measurement instruments

The participants completed a survey containing questions on their demographic characteristics and sexual activities for the previous 6 months and standardized scales, such as the Barratt Impulsiveness Scale-11 (Patton et al., 1995), Buss-Perry Aggression questionnaire (Buss and Perry, 1992), Beck Depression Inventory (Beck et al., 1996), Beck Anxiety Inventory (Beck et al., 1996), Sexual Addiction Screening Test-R (SAST-R; Carnes et al., 2010), and Hypersexual Behavior Inventory (HBI; Reid et al., 2011; Table 1). The questions on sexual behavior were age of first sexual intercourse and current sexual relationship status. An *exclusive sexual situation* was defined as a relationship in which only two individuals engage in sexual intercourse exclusively with each other. A *nonexclusive sexual relationship* was defined as the maintaining of multiple sexual relationships with several different sexual partners without maintaining any sort of intimacy in the relationship.

**Table 1:** **Subject characteristics**.

	**Controls (*n* = 22)**	**Individuals with PHB (*n* = 23)**	***T*-values**
Age	26.3 (3.4)	26.1 (4.1)	
Marital Status^a^			
Single	50.0	47.8	
In a relationship	41.0	43.5	
Engaged/Married	9.0	8.7	
Age of first sexual intercourse	20.3 (3.7)	16.7 (5.9)	2.44^*^
Sexual Relationship Status^a^			
Exclusive	50.0	30.4	
Nonexclusive	13.6	56.5	
Not sexually active	36.4	13.1	
Number of sex partners^b^	2.5 (3.5)	20.9 (27.5)	3.11^**^
Frequency of sexual intercourse per week^b^	0.5 (0.7)	3.7 (2.6)	5.58^***^
Frequency of masturbation per week^b^	1.7 (0.9)	5.1 (3.2)	4.80^***^
Frequency of viewing pornography per week^b^	2.3 (0.6)	5.5 (2.7)	5.42^***^
Barratt Impulsiveness Scale-11 Score	50.9 (5.5)	52.6 (6.9)	0.91
Buss-Perry Aggression questionnaire Score	37.4 (6.9)	51.5 (16.6)	3.68^**^
Beck depression inventory score	5.3 (1.6)	7.5 (4.8)	1.53
Beck anxiety inventory score	7.3 (6.4)	8.5 (8.3)	2.04^*^
Sexual addiction screening test-R score	0.5 (0.9)	11.3 (3.3)	14.82^***^
Hypersexual behavior inventory score	26.9 (13.5)	54.4 (7.3)	8.55^***^

PHB, Problematic Hypersexual Behavior.

a Data are represented as percentage.

b During 6 months; Data are represented as means (standard deviation).

* *p < 0.05*,

** *p < 0.01*,

*** *p < 0.001*.

The questions on sexual activity-related characteristics included the frequency of sexual intercourse per week, the frequency of masturbation per week, the frequency of viewing pornography per week, and the number of total sexual partners in the past 6 months. Furthermore, the SAST-R (Carnes et al., 2010) and HBI (Reid et al., 2011) were used to assess the degree of PHB in the participants. The SAST-R consists of 20 questions designed to assess the degree of sexual addiction. The score ranges from 0 to 20 points, with higher scores indicating more severe sexual addiction. The HBI is comprised of 19 questions, and the score ranges from 19 to 95. A total score of 53 or higher is indicative of a hypersexual disorder. The internal consistencies (Cronbach's α coefficient) of the SAST-R and HBI are 0.91 and 0.96, respectively (Carnes et al., 2010; Reid et al., 2011).

### Experimental stimuli and experimental paradigm

A prestudy was conducted on 130 men with normal sexual functions who did not participate in the fMRI experiment in order to select the sexual and nonsexual stimuli for the fMRI study (File S1). The visual stimuli consisted of 20 photos that were collected from the International Affective Picture System (6 photos; Lang et al., 2008) and Internet websites (14 photos). The sexual stimuli consisted of photographs depicting naked women and sexual activity. In addition, 20 photos that did not induce any sexual desire were chosen as the nonsexual stimuli. They were matched with the sexual stimuli for their level of pleasantness. The nonsexual stimuli displayed highly arousing scenes, such as water sport activities, celebration of a winning victory, and skiing. These stimuli were chosen in order to identify the brain activity that was solely related to sexual desire by ruling out activity that resulted from feelings of pleasantness and general arousal.

**Figure 1:**
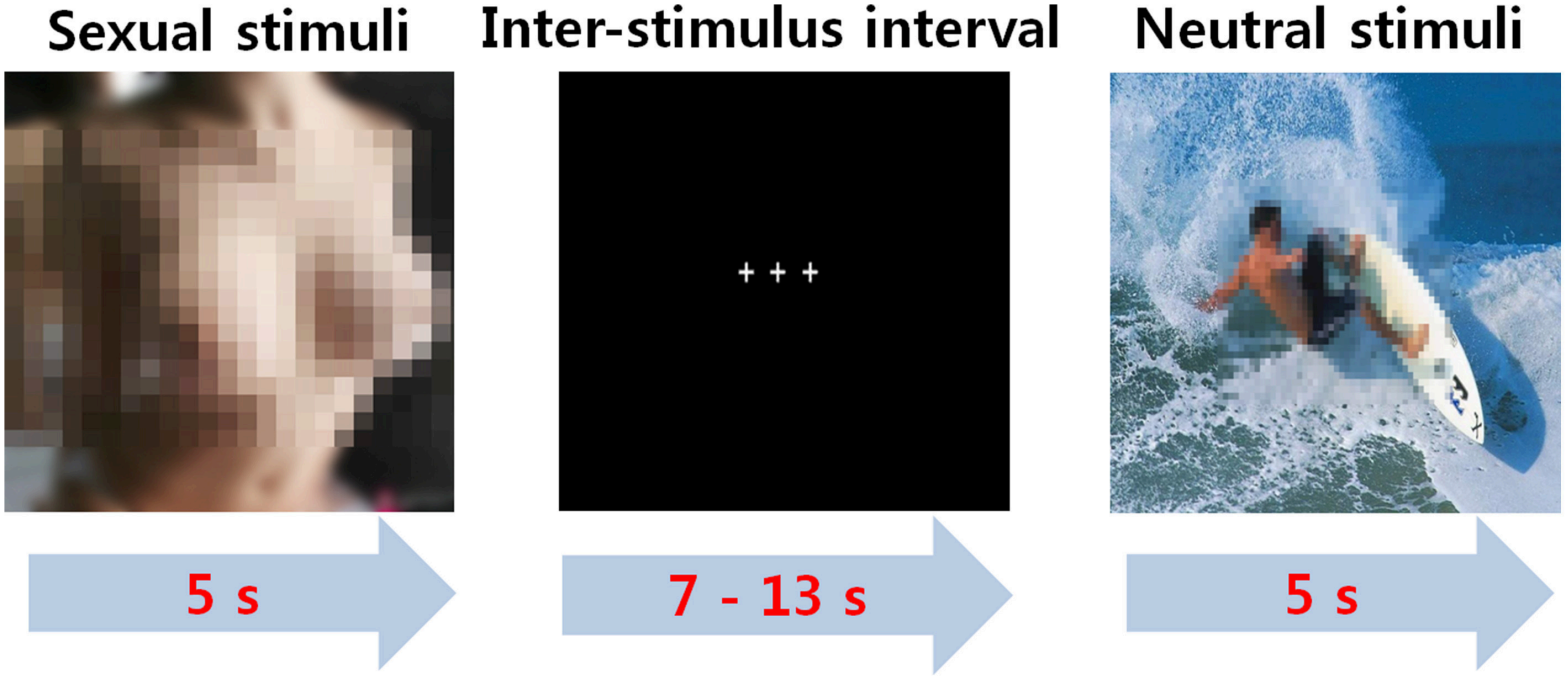
**The event-related paradigm for sexual desire**.

For the fMRI experimental paradigm, brief instructions about the experiment were given for 6 s at the beginning of the experiment, which was followed by the random presentation of either sexual or nonsexual stimuli for 5 s each. Each interstimulus interval was 7–13 s (average, 10 s) to help the participant to return to their baseline state. To keep the participants focused on the stimuli, they were asked to press the response button when an unexpected target was presented for approximately 500 ms for a total of 12 times during any interval. The total time required for the experiment was 8 min and 48 s (Figure 1).

After completing the fMRI experiment, the participants watched the same stimuli that were presented in the fMRI experiment, and they were required to respond to the following three questions for a psychological assessment. First, they were asked to respond “yes” or “no” when asked whether they felt sexual desire when they visualized each stimulus. Second, they were required to rate their sexual desire on a five-point Likert scale ranging from 1 (least intense) to 5 (most intense). Third, the participants' subjective ratings on the dimensions of valence and arousal to each stimulus were determined according to a seven-point Likert scale. The ratings were formulated on two dimensions. Valence, which was positive or negative, ranged from very negative at 1 to very positive at 7, and emotional arousal ranged from calm at 1 to excited/aroused at 7. Finally, the participants were required to report any other emotions that they experienced besides sexual desire during their exposure to each stimulus.

### Image acquisition

Image acquisition was performed with a 3.0 T Philips magnetic resonance scanner (Philips Healthcare, Best, The Netherlands). A single-shot echo-planar imaging fMRI scanning method [imaging variables: repetition time (TR) = 2,000 ms, echo time (TE) = 28 ms, slice thickness = 5 mm with no gap, matrix = 64 × 64, field of view (FOV) = 24 × 24 cm, flip angle = 80°, and in-plane resolution = 3.75 mm] was used to acquire 35 continuous slices of blood oxygen level-dependent (BOLD) images. T1-weighted anatomical images were obtained with a 3-dimensional fluid-attenuated inversion recovery sequence (*TR* = 280, *TE* = 14 ms, flip angle = 60°, *FOV* = 24 × 24 cm, matrix = 256 × 256, and slice thickness = 4 mm).

### Statistical analyses

In order to investigate the behavioral and neural responses that were based solely on sexual desire, the imaging and psychological data for the three pictures that induced other emotions, such as disgust, anger, or surprise, other than sexual arousal were excluded from the data analysis. Independent *t*-tests of the frequencies and intensities of sexual desire between the two groups were performed using SPSS 22 (IBM Corporation, Armonk, NY, USA). The frequency of sexual desire was considered the number of stimuli for which each participant experienced sexual desire from among the total 20 sexual stimuli, and the intensity of sexual arousal was the average level of subjective sexual desire for the 20 erotic pictures.

SPM8 (Wellcome Department of Imaging Neuroscience, London, UK) was used to analyze the fMRI data. In the preprocessing stage, MRI image acquisition was performed in the following order: slice-timing correction for interleaved acquisition, motion correction, and spatial normalization onto a standard template provided by the Montreal Neurological Institute (MNI). Subsequently, the normalized images were smoothed with an 8-mm Gaussian kernel.

After completing the preprocessing, design matrices with two conditions (sexual condition and nonsexual condition) were created for each participant to identify the areas with sexual desire-related activation. Individual first-level analyses of the comparisons of sexual condition minus nonsexual condition were used for a random effects analysis, and mean images were created for each subject. One-sample *t*-tests on the mean images were used to assess the significant group effects in each group in the contrast images created in the individual analyses. Two-sample *t*-tests were conducted to identify the differences between the two groups for the brain responses in the sexual condition relative to the nonsexual condition. Additionally, correlational analyses were conducted only in the PHB group to determine the regions of activation that correlated with the severity of hypersexuality according to the SAST-R. Because the variance of the questionnaire scores might have been too low to reveal more significant correlations in the control group, correlational analyses were not conducted in the control group. P values less than 0.05 (False Discovery Rate, corrected, cluster size ≥ 20) or 0.001 (uncorrected, cluster size ≥ 20) were considered significant for brain activity as these levels are generally accepted in fMRI studies. All of the coordinates of the activated voxels are shown as MNI coordinates in **Tables 3**, **4**.

The percent signal change was extracted from the Regions of Interest (ROIs) based on the results of the between-group and correlation analyses [i.e., bilateral thalamus, right dorsolateral prefrontal cortex (DLPFC), left caudate nucleus, right supramarginal gyrus, and right dorsal anterior cingulate gyrus] with MarsBaR (http://www.sourceforge.net/projects/marsbar). The ROIs were created by placing a 5-mm sphere around the coordinates reported in **Tables 3**, **4**. In order to examine the temporal characteristics of the hemodynamic responses, the BOLD signal time course was also extracted from the ROIs during the presentation of each sexual stimulus (total of 12 s; 5 s and 7 s thereafter) for all of the participants. The time courses were then averaged across the participants in each group.

As a follow-up test of correlation to calculate the correlation coefficient, the relationships between the scores on the SAST-R and HBI and the percent signal changes in the ROIs based on the results of the correlation analysis (**Table 4**) were analyzed in the PHB group with SPSS 22.

## Results

### Results of the psychological assessments

Of the 20 healthy control subjects, only two reported other emotions besides sexual arousal in response to the three sexual stimuli. One participant in the control group reported that two sexual stimuli among the 20 sexual stimuli induced disgust and anger, while the other participant in the control group rated that one sexual picture induced surprise. The three sexual pictures that induced feelings other than sexual arousal were excluded from the data analysis.

An independent *t*-test indicated no group differences in the dimensions of valence and arousal in response to sexual cues [valence: *t*_(43)_ = 0.14, *p*>0.05, Cohen's *d* = 0.042; arousal: *t*_(43)_ = 0.30, *p*>0.05, Cohen's *d* = 0.089]. Additionally, the percentage of sexual stimuli among the 20 erotic pictures that evoked sexual desire showed that the PHB group felt sexual desire more frequently than the control group during exposure to sexual stimuli [*t*_(43)_ = 3.23, *p* < 0.01, Cohen's *d* = 0.960]. The intensity of sexual arousal showed that the PHB group experienced more intense sexual arousal than the control group in response to sexually stimulating photos [*t*_(43)_ = 14.3, *p* < 0.001, Cohen's *d* = 4.26]. The results of the psychological assessments are shown in Table 2.

**Table 2:** **Psychological assessment results**.

	**Controls *(n* = 22)**	**Individuals with PHB *(n* = 23)**	***t***
Frequency of sexual desire (%)^a^	50.2 (36.7)	81.6 (28.0)	3.23^**^
Intensity of sexual desire^b^	0.5 (0.5)	2.6 (0.5)	14.30^***^
Valence	4.45 (1.41)	4.51 (1.45)	0.14
Arousal	3.53 (1.72)	3.69 (1.70)	0.30

*PHB, Problematic Hypersexual Behavior*.

*Data are represented as means (standard deviation)*;

** *p < 0.01*,

*** *p < 0.001*.

a *Represented as percentage of sexual stimuli that evoked sexual desire among 20 erotic pictures*.

b *Degree of sexual desire triggered by the sexual cues on a five-point Likert scale*.

### fMRI results

In the PHB group, activation was observed in the bilateral middle/inferior frontal gyri [Brodmann area (BA) 9], cuneus/precuneus (BA 7, 18, and 19), striatum, thalamus, and cingulate gyri (BA 24 and 32) in response to sexual stimuli compared with nonsexual stimuli. In the control group, activation was displayed in the bilateral middle/inferior frontal gyri (BA 9), cuneus/precuneus (BA 7, 18, and 19), striatum, thalamus, and left cingulate gyrus (BA 24) (corrected False Discovery Rate, *p* < 0.05).

**Figure 2:**
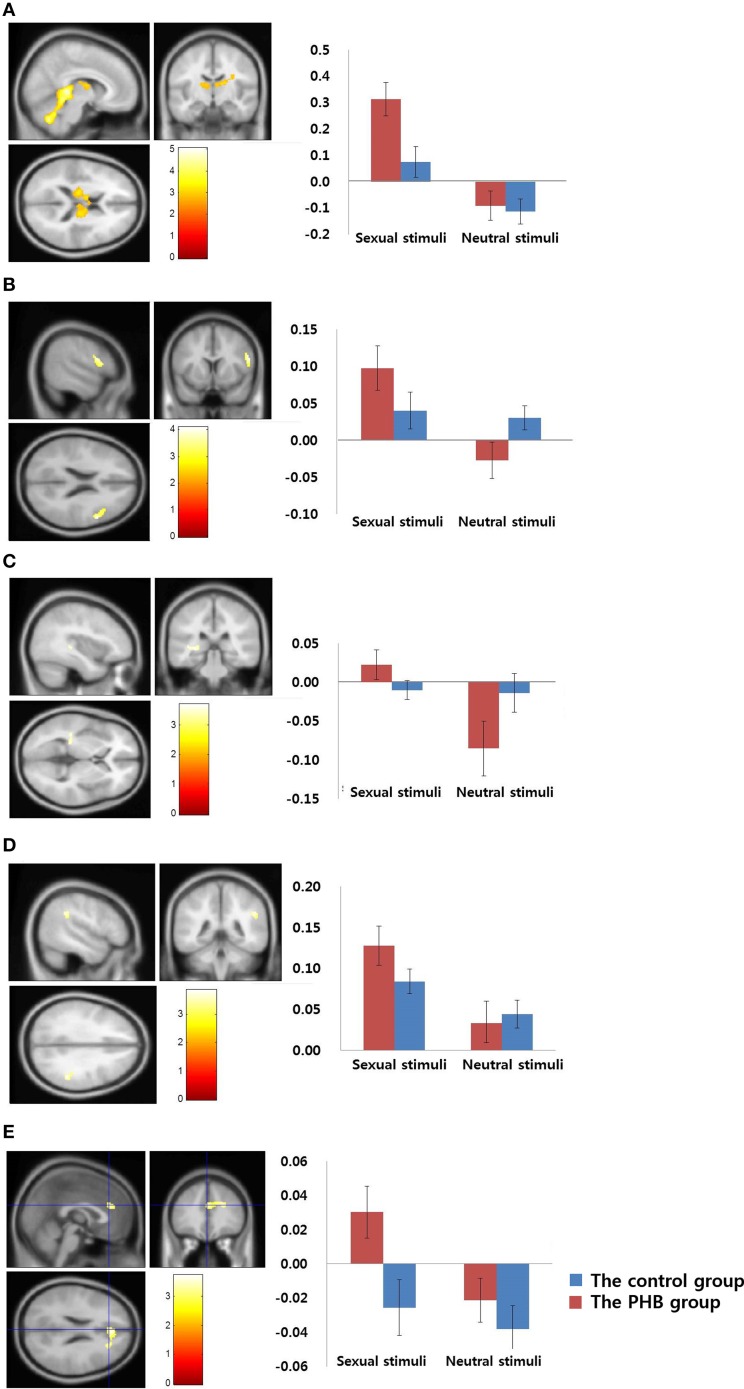
**Results of the between-group analysis**. **(A)** Bilateral thalamus (MNI coordinate; *x* = 6, *y* = −36, *z* = 4) **(B)** Right dorsolateral prefrontal cortex (MNI coordinate; *x* = 56, *y* = 10, *z* = 22) **(C)** Left caudate nucleus (MNI coordinate; *x* = −38, *y* = −32, *z* = 2) **(D)** Right supramarginal gyrus (MNI coordinate; *x* = 50, *y* = −42, *z* = 32) **(E)** Right dorsal anterior cingulate gyrus (MNI coordinate; *x* = 24, *y* = −16, *z* = 34). Results of the comparisons of activation in sexual stimuli minus nonsexual stimuli between the PHB and control groups (*p* < 0.05, False Discovery Rate, corrected). The control group and the PHB group are represented as blue and red, respectively. The y-axis shows the percent signal change and the error bars represents Standard Error of the Mean.

**Figure 3:**
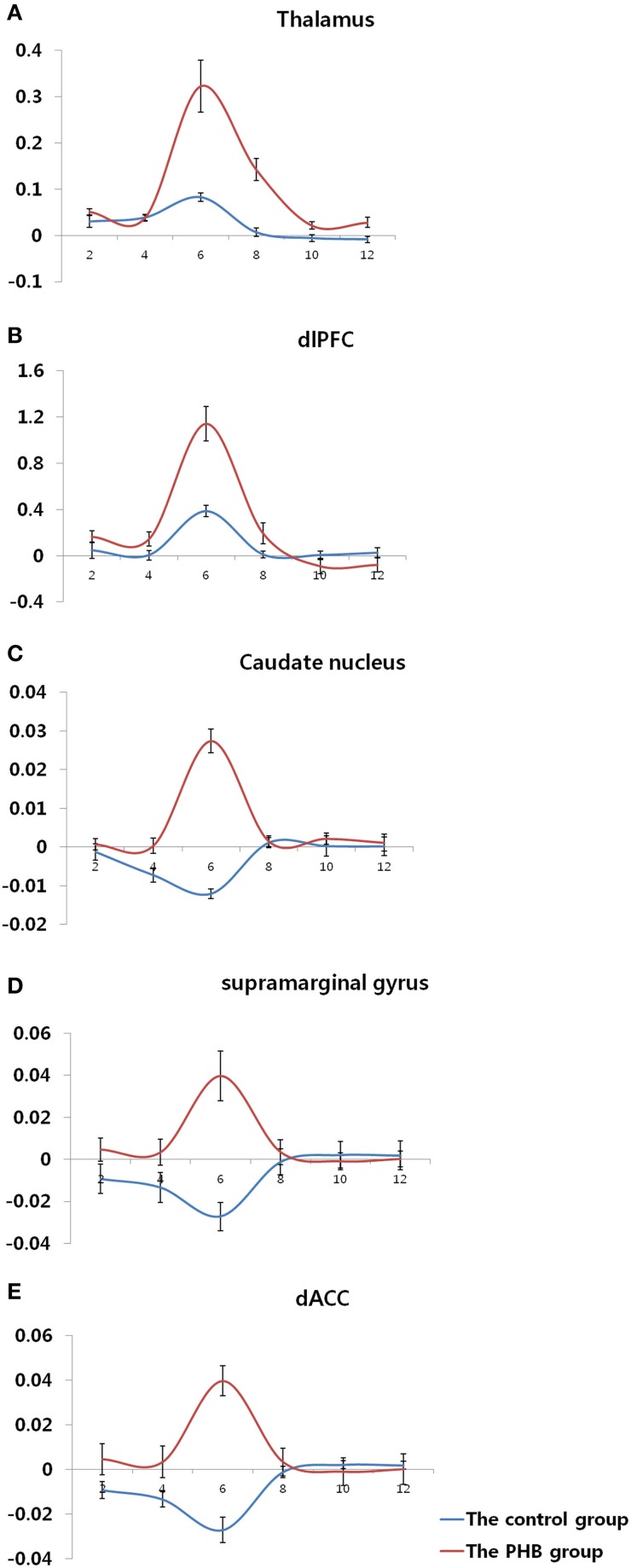
**Time course of the hemodynamic responses in each region of interest**. **(A)** Bilateral thalamus (MNI coordinate; *x* = 6, *y* = −36, *z* = 4) **(B)** Right dorsolateral prefrontal cortex (MNI coordinate; *x* = 56, *y* = 10, *z* = 22) **(C)** Left caudate nucleus (MNI coordinate; *x* = −38, *y* = −32, *z* = 2) **(D)** Right supramarginal gyrus (MNI coordinate; *x* = 50, *y* = −42, *z* = 32) **(E)** Right dorsal anterior cingulate gyrus (MNI coordinate; *x* = 24, *y* = −16, *z* = 34). The y-axis and x-axis display the percent signal change and time (s), respectively, and the error bars represent the Standard Error of the Mean.

In the between-group analysis, the PHB group exhibited greater activation in the right dorsal anterior cingulate cortex (dACC; BA 24 and 32), bilateral thalami, left caudate nucleus, right DLPFC (BA 9, 46), and right supramarginal gyrus (BA 40) relative to the activation in the control group during exposure to sexual stimuli compared with nonsexual stimuli. No brain regions in the control group showed greater activation than in the PHB group. All of the coordinates for the activated voxels are shown as MNI coordinates in Tables 3, 4. Figure 2 shows the percent signal changes in the control and PHB groups in each experimental condition (that is, sexual and nonsexual conditions) for the selected ROIs, and Figure 3 displays the mean time series for each group of the percent signal changes at each time point in the ROIs during the presentation of each sexual stimulus (total of 12 s; 5 and 7 s thereafter) based on the results of the between group analysis.

**Table 3:** **Brain regions identified by the group analysis**.

**Brain regions**	***P***	**No. of Voxels in cluster**	**Cluster *z* Score**	**x, y, z MNI coordinates**		
**INDIVIDUALS WITH PHB < CONTROLS**						
No region						
**INDIVIDUALS WITH PHB > CONTROLS**						
Bilateral thalamus	0.034	129	4.25	6	−36	4
Right dorsolateral prefrontal	0.035	190	3.75	56	10	22
cortex	0.041		3.42	54	16	14
(BA 9, 46)	0.042	22	3.32	46	16	26
Right supramarginal gyrus	0.037	78	3.56	50	−42	32
(BA 40)	0.049		3.01	42	−38	32
Right dorsal cingulate gyrus	0.037	79	3.54	24	16	34
(BA 24, 32)	0.043		3.28	16	10	40
Left caudate nucleus	0.038	53	3.51	−38	−32	2

MNI, Montreal Neurological Institute; PHB, Problematic Hypersexual Behavior; BA, Brodmann area.

Comparison of brain activation between the two groups (PHB group vs. control group) during the sexual condition compared to the nonsexual condition (corrected False Discovery Rate, p < 0.05).

**Table 4:** **Brain regions identified in the correlational analysis in the PHB group during exposure to sexual stimuli**.

**Brain region**	***p***	**No. of Voxels in cluster**	**Cluster z Score**	**x, y, z MNI coordinates**		
Right thalamus	0.001	80	4.25	4	−32	6
Right dorsolateral prefrontal cortex	0.001	22	3.77	56	8	22

*PHB, Problematic Hypersexual Behavior; MNI, Montreal Neurological Institute*.

*Brain regions significantly correlated with Sexual Addiction Screening Test-R (SAST–R) scores during the sexual condition in the PHB group (p < 0.001, uncorrected)*.

**Figure 4:**
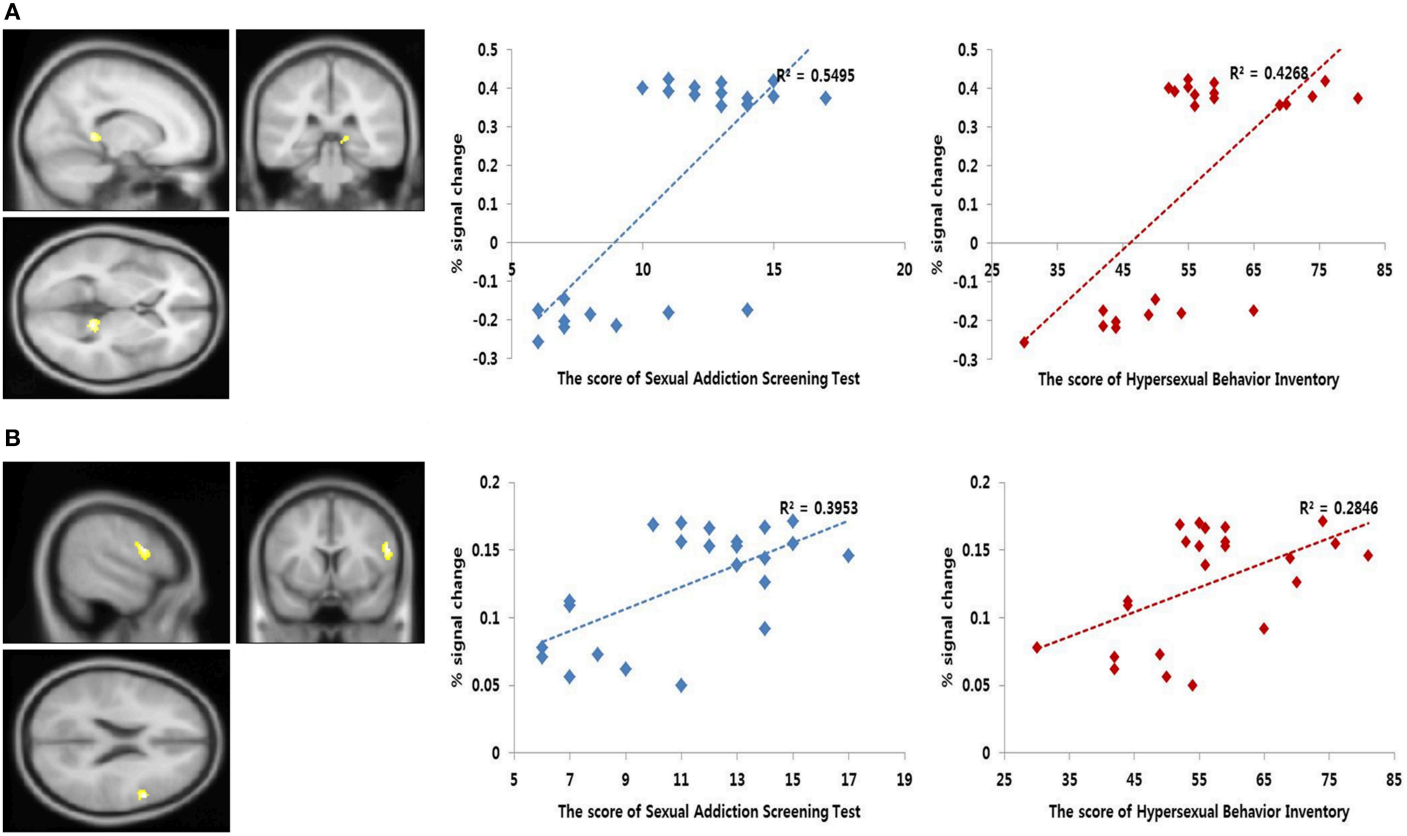
**Results of the correlation analysis**. Left, functional magnetic resonance imaging (fMRI) correlational analysis. The regions showing significant correlation between the brain activity during sexual desire and the Sexual Addiction Screening Test-R (SAST-R) scores (*p* < 0.001, uncorrected). Right, linear relationship between the percent signal changes extracted from each area and sexual severity scores [i.e., SAST-R and Hypersexual Behavior Inventory (HBI) scores]. The x-axis shows the sexual severity scores, and the y-axis represents the percent signal change. **(A)** Bilateral thalamus (MNI coordinate; *x* = 4, *y* = −32, *z* = 6) **(B)** Right dorsolateral prefrontal cortex (MNI coordinate; *x* = 56, *y* = 8, *z* = 22).

The correlation analysis of the regions that were related to the SAST-R score demonstrated that the right thalamus and DLPFC (BA 9) were correlated with the SAST-R scores (*p* < 0.001, uncorrected) in the PHB group during the exposure to sexual stimuli, as shown in Table 4. The results of the follow-up analysis showed that the percent signal change that was extracted from the right thalamus and DLPFC correlated significantly with the severity of hypersexuality, as shown in Figure 4. The percent signal changes in the right thalamus and right DLPFC correlated positively with the SAST-R scores in the PHB group during exposure to sexual stimuli (right thalamus: *r* = 0.74, *n* = 23, *p* < 0.01; right DLPFC: *r* = 0.63, *n* = 23, *p* < 0.01). In addition, the percent signal changes in the right DLPFC and right thalamus were positively related to the HBI scores in the PHB group (right thalamus: *r* = 0.65, *n* = 23, *p* < 0.01; right DLPFC: *r* = 0.53, *n* = 23, *p* < 0.01), as shown in Figure 4.

## Discussion

The present study examined whether there was a difference in the levels of sexual desire between individuals with PHB and healthy controls and, if so, whether this difference was related to functional alterations in the neural substrates of sexual desire in these individuals. As predicted, the PHB group showed significantly heightened levels of sexual desire and altered activation in the PFC and subcortical areas compared to controls. These results suggested that the functional changes in the neural circuitry that mediates cue-induced desire for sexual behavior were similar to those in response to cue presentation in individuals with substance addiction or behavioral addiction (Garavan et al., 2000; Tapert et al., 2003; Crockford et al., 2005; Franklin et al., 2007; Ko et al., 2009; McClernon et al., 2009). Voon et al. (2014) reported abnormal desire and functional changes in regions associated with heightened desire in individuals with compulsive sexual behavior. We replicated and extended these results by investigating the times series of the activation during the total 12 s in the areas associated with sexual desire.

As hypothesized, the analyses of the results of the psychological assessments showed that the PHB group exhibited more frequent sexual desire than the control group during exposure to sexual stimuli, which suggested that this group had a lower threshold for sexual desire. When sexual desire was induced, the PHB group showed a higher intensity of sexual desire than the control group did. This result was consistent with previous findings on individuals with PHB group (Laier et al., 2013; Laier and Brand, 2014; Voon et al., 2014), especially demonstrate that the desire for pornography might play a key role in cybersex addiction.

The results on the brain responses to sexual stimuli dovetail nicely with previous neuroimaging findings that indicated that activity is observed in the brain regions involved in sexual wanting or motivation/anticipation, as well as sexual liking or arousal/consummation, when all of the participants are exposed to sexual stimuli (Georgiadis and Kringelbach, 2012). The results of the group comparisons of the brain imaging revealed altered activation in the right DLPFC (BA 9) and subcortical regions, including the right dACC (BA 24 and 32), left caudate nucleus, right supramarginal gyrus (BA 40), and right thalamus, and these alterations might be associated with the behavioral characteristics of the PHB group. In addition to brain activation, we examined a time series of the hemodynamic responses in these areas during and after the arousal of sexual desire in these areas.

Among these regions, the left caudate nucleus and right ACC (BA 24 and 32) and the right DLPFC are assumed to be associated with the motivational component of sexual desire. The involvement of the caudate nucleus in motivation and reward processing might account for its response to sexual stimuli (Delgado, 2007). The dorsal striatum is activated during reward anticipation (Delgado, 2007), which possibly reflects the desire that is associated with such anticipation. In a study of the neural responses associated with pornography consumption, frequent activation as a result of pornography exposure might result in the wearing down and downregulation of the striatum, including the caudate nucleus, in healthy controls (Kühn and Gallinat, 2014). However, in the current study, greater activation was observed in the caudate nucleus in the PHB group, even though the PHB group watched pornography more often. These differences between the results of the present study and those of Kühn and Gallinat (2014) might be explained by the difference in the participants. That is, in contrast to the use of healthy male adults in the previous study, our study was conducted on individuals with PHB. Accumulating evidence suggests that the caudate nucleus is important for stimulus-response habit learning and the maintenance of addictive behavior (Vanderschuren and Everitt, 2005). The activation of the caudate nucleus in this study might suggest that sexual cue-reactivity is established after repeated exposure to sexual experience.

The dACC is known to be related to the motivational mechanisms of sexual desire (Redouté et al., 2000; Arnow et al., 2002; Hamann et al., 2004; Ferretti et al., 2005; Ponseti et al., 2006; Paul et al., 2008). Our findings of dACC activation suggest that it has a role in sexual desire, and these results were similar to those of a study on desire-related neural activity in subjects with compulsive sexual behaviors (Voon et al., 2014). In addition, the dACC is known to be important in the initial processing of goal-oriented behavior by engaging in conflict monitoring between the urge for behavioral expression and the suppression of that urge (Devinsky et al., 1995; Arnow et al., 2002; Karama et al., 2002; Moulier et al., 2006; Safron et al., 2007). Neuroanatomically, the dACC projects to the DLPFC and parietal lobe (Devinsky et al., 1995; Pizzagalli et al., 2001). In this study, the activation in the dACC in the PHB group might reflect internal conflict between the urge to express sexual impulses as actions and the urge to suppress the impulses due to situational factors during the presentation of sexual stimuli.

The activation of the supramarginal gyrus is associated with increased attention to targets that are perceived as sexual cues (Redouté et al., 2000; Stoléru et al., 2012). Previous studies have proposed that the increased attention to sexual stimuli plays an important role in maintaining sexual desire (Barlow, 1986; Janssen and Everaerd, 1993) and is related to sexual sensation seeking (Kagerer et al., 2014). In the current study, the supramarginal activation could reflect the greater attention that was paid by PHB subjects to sexual stimuli and that could result in the higher levels of sexual desire compared with the control group.

Among the regions that were significantly activated in the between group results, the DLPFC and thalamus directly correlated with the severity of sexual addiction in the PHB subjects. We observed greater thalamus activation, which was in line with previous findings of studies on sexual arousal (Redouté et al., 2000; Moulier et al., 2006). According to previous studies on sexual desire, the activation of the thalamus is related to the physiological responses (i.e., readiness for sexual activity) that are induced by sexual desire and is positively correlated with penile erection (MacLean and Ploog, 1962; Redouté et al., 2000; Moulier et al., 2006). Interestingly, we also found a higher and wider hemodynamic pattern in the thalamus compared with that in controls. This higher and wider hemodynamic response might indicate that sexual arousal was stronger and prolonged in the individuals with PHB.

Similar to the findings of studies on neural activity in individuals with addiction during cue-induced desire, we found altered PFC function in the PHB group. The PFC plays a critical role in future planning and working memory (Bonson et al., 2002). Neuroanatomically, the PFC is interconnected to various areas, including the dACC, caudate nucleus, and parietal lobe (Devinsky et al., 1995; Pizzagalli et al., 2001; Goldman-Rakic and Leung, 2002). Previous studies on addiction have demonstrated that dysfunction of this network, including the PFC, is related to the PFC's regulation of limbic reward regions and its involvement in higher-order executive function, including self-control, salience attribution, and awareness (Goldman-Rakic and Leung, 2002; Feil et al., 2010; Goldstein and Volkow, 2011; Kühn and Gallinat, 2014). In particular, these studies have identified the disrupted function of DLPFC as an impairment in salience attribution, which results in symptoms, such as the abnormally increased sensitivity to an addictive cue as in substance and addicted behaviors and decreased interest to normal-rewarding stimuli (Goldman-Rakic and Leung, 2002; Goldstein and Volkow, 2011). In the current study, the observation of greater DLPFC activation in the PHB group compared to the control group might reflect excessive salience attribution to sexual cues.

In summary, the PHB group showed greater sexual desire that was associated with altered brain activity. These findings indicate that the PHB group might pay excessive attention to sexual stimuli and that it might have an automatic response because the conditional response to sexual stimuli could not be mediated properly. The limitations of the present study were as follows. First, the race of the subjects was Asian. Second, this study involved only heterosexual male subjects, and future studies involving females and homosexual male subjects should be helpful in better understanding PHB. PHB subjects with co-occurring mental disorders were not enrolled in the present study, thus ensuring the investigation of neural dysfunction based solely on PHB. However, according to a study by Weiss (2004), 28% of males with PHB suffer from major depressive disorder. Taking these factors together limit the generalizability of the study results to the broader universal population. Finally, the two groups may have differed with respect to self-awareness and/or emotional sensitivity due to the treatment of the PHB participants. We tried to decrease the differences between the control and PHB groups by matching for important demographic variables, including age, education level, and handedness, for comparison purposes and by applying strict exclusion criteria, such as the presence of psychiatric disorders and the current use of psychotropic medication, to both groups. Next, we plan to examine how variables that are related to treatment period or treatment type affect the emotional responses, including responses to sexual cues, of individuals with PHB.

Despite these limitations, the results of this study contribute significantly to the literature and have significant implications for future research. We identified specific brain regions that were directly associated with sexual desire and the temporal changes in the activities of these regions among subjects with PHB. Like brain imaging studies on substance and behavior addiction, PHB was related to functional changes in the PFC and subcortical areas, even without the neurotoxicity of drugs. Our results are therefore useful for characterizing the behaviors and associated neural mechanisms of individuals with PHB, and go a step beyond the descriptions of characteristics as in previous studies.

## Funding

This work was supported by the Korea Basic Science Institute (No. E35600) and research fund of 2014 Chungnam National University.

### Conflict of interest statement

The authors declare that the research was conducted in the absence of any commercial or financial relationships that could be construed as a potential conflict of interest.
