# Supplementary material for: Neural Substrates of Sexual Desire in Individuals with Problematic Hypersexual Behavior
Source: Front Behav Neurosci. 2015 Nov 30;9:321. doi: 10.3389/fnbeh.2015.00321 (PMC4663274; doi:10.3389/fnbeh.2015.00321)
Supplement: Supplementary file 1 [file DataSheet1.DOCX]

**Supplementary materials**

**Methods**

A group experiment was conducted on 130 men with normal sexual functions (those who did not participate in the fMRI experiment) to select the sexual and non-sexual stimuli for the fMRI study. They were presented with 237 photos (116 erotic pictures and 121 non-erotic pictures) obtained from the International Affective Picture System (IAPS) [36] and internet searches. The each picture was presented for 3 seconds, and the participants had been instructed to look at the picture during the entire period it was presented. After presentation of the picture, the participants were asked the following questions to assess suitability, effectiveness, positiveness, and arousal: ‘Does this picture provoke the sexual desire? Please answer yes or no’, ‘How much does this picture increase your sexual desire? Please rate the intensity on below scale ranging from 1 (least intense) to 7 (most intense)’, ‘How pleasant is this picture? Please rate the pleasantness on below scale ranging from 1 (not at all) to 7 (extremely)’, ‘How excited is this picture? Please rate the excitement on below scale ranging from 1 (not at all) to 7 (extremely)’. After the rating interval was over, the next picture was displayed. This continued until all 237 pictures were viewed and rated. As a result of the pre-study, 20 sexual pictures were selected. Percentage of suitability and mean scores of, effectiveness, positiveness, and arousal to sexual stimuli was 86.14 (SD = 5.64), 5.59 (SD = 0.44), 5.23 (SD = .36) and 5.17 (SD = .31), respectively. Twenty non-sexual stimuli matched with the sexual stimuli for their level of pleasantness (M = 5.10 SD = .31) and arousal 4.96 (SD = .38) were selected (t = -1.18, p > 0.05; t = -1.99, p > 0.05). Mean scores of suitability, effectiveness, positiveness, and arousal to non- sexual stimuli was 0 (SD = 0), 0 (SD = 0), 5.10 (SD = .31) and 4.96 (SD = .38), respectively.

Table S1. Diagnostic criteria for individuals with PHB

|  | Diagnostic criteria |
| --- | --- |
| Sexual addiction (Carnes et al., 2010) | Three or more of the following symptoms:   1. Recurrent failure to resist impulses to engage in specific sexual behavior 2. Frequent engaging in sexual behaviors to a greater extent or over a longer period of time than intended 3. Persistent desire or unsuccessful efforts to stop, reduce, or control sexual behaviors 4. Inordinate amount of time spent in obtaining sex, being sexual, or recovering from sexual experience 5. Preoccupation with sexual behavior or preparatory activities 6. Frequent engaging in sexual behavior when expected to fulfill occupational, academic, domestic, or social obligations 7. Continuation of sexual behavior despite knowledge of having a persistent or recurrent social, financial, psychological, or physical problem that is caused or exacerbated by the behavior 8. Need to increase the intensity, frequency, number, or risk of sexual behaviors to achieve the desired effect, or diminished effect with continued sexual behaviors at the same level of intensity, frequency, number, or risk 9. Giving up or limiting social, occupational, or recreational activities because of sexual behavior 10. Distress, anxiety, restlessness, or irritability if unable to engage in sexual behavior |
| A proposed criteria of hypersexual disorder for DSM-V (Kafka, 2010) | A. Over a period of at least 6 months, recurrent and intense sexual fantasies, sexual urges, or sexual behaviors in association with 3 or more of the following 5 criteria:  A1. Time consumed by sexual fantasies, urges or behaviors repetitively interferes with other important (non-sexual) goals, activities and obligations.  A2. Repetitively engaging in sexual fantasies,  urges or behaviors in response to dysphoric  mood states (e.g., anxiety, depression,  boredom, irritability).  A3. Repetitively engaging in sexual fantasies, urges or behaviors in response to stressful life events.  A4. Repetitive but unsuccessful efforts to control or significantly reduce these sexual fantasies, urges or behaviors.  A5. Repetitively engaging in sexual behaviors while disregarding the risk for physical or emotional harm to self or others.  B. There is clinically significant personal distress or impairment in social, occupational or other important areas of functioning associated with the frequency and intensity of these sexual fantasies, urges or behaviors.  C. These sexual fantasies, urges or behaviors are not due to the direct physiological effect of an exogenous substance (e.g., a drug of abuse  or a medication)  Specify if: Masturbation, Pornography, Sexual Behavior with Consenting Adults, Cybersex, Telephone Sex, Strip Clubs |
